# Clinical outcome of CIDP one year after start of treatment: a prospective cohort study

**DOI:** 10.1007/s00415-021-10677-5

**Published:** 2021-06-26

**Authors:** S. R. M. Bus, M. C. Broers, I. M. Lucke, C. Bunschoten, G. G. A. van Lieverloo, M. E. Adrichem, R. van Veen, L. Wieske, H. F. Lingsma, H. S. Goedee, W. L. van der Pol, I. N. van Schaik, P. A. Van Doorn, B. C. Jacobs, F. Eftimov, for the ICOS Consortium

**Affiliations:** 1 Department of Neurology, Amsterdam University Medical Center, University of Amsterdam, Amsterdam Neuroscience, Amsterdam, The Netherlands; 2 Department of Neurology, Erasmus MC, University Medical Center, Rotterdam, The Netherlands; 3 Department of Public Health, Erasmus MC, University Medical Center, Rotterdam, The Netherlands; 4 Department of Neurology, Brain Center Rudolf Magnus, University Medical Center Utrecht, Utrecht, The Netherlands; 5 Department of Immunology, Erasmus MC, University Medical Center, Rotterdam, The Netherlands; 6 Spaarne Gasthuis, Haarlem, The Netherlands

**Keywords:** Chronic inflammatory demyelinating polyradiculoneuropathy, CIDP, Intravenous immunoglobulin, Corticosteroids

## Abstract

**Objective:**

To assess clinical outcome in treatment-naive patients with chronic inflammatory demyelinating polyradiculoneuropathy (CIDP).

**Methods:**

We included adult treatment-naive patients participating in the prospective International CIDP Outcome Study (ICOS) that fulfilled the European Federation of Neurological Societies/Peripheral Nerve Society (EFNS/PNS) diagnostic criteria for CIDP. Patients were grouped based on initial treatment with (1) intravenous immunoglobulin (IVIg), (2) corticosteroid monotherapy or (3) IVIg and corticosteroids (combination treatment). Outcome measures included the inflammatory Rasch-built overall disability scale (I-RODS), grip strength, and Medical Research Council (MRC) sum score. Treatment response, treatment status, remissions (improved and untreated), treatment changes, and residual symptoms or deficits were assessed at 1 year.

**Results:**

Forty patients were included of whom 18 (45%) initially received IVIg, 6 (15%) corticosteroids, and 16 (40%) combination treatment. Improvement on ≥ 1 of the outcome measures was seen in 31 (78%) patients. At 1 year, 19 (48%) patients were still treated and fourteen (36%) patients were in remission. Improvement was seen most frequently in patients started on IVIg (94%) and remission in those started on combination treatment (44%). Differences between groups did not reach statistical significance. Residual symptoms or deficits ranged from 25% for neuropathic pain to 96% for any sensory deficit.

**Conclusions:**

Improvement was seen in most patients. One year after the start of treatment, more than half of the patients were untreated and around one-third in remission. Residual symptoms and deficits were common regardless of treatment.

**Supplementary Information:**

The online version contains supplementary material available at 10.1007/s00415-021-10677-5.

## Introduction

Chronic inflammatory demyelinating polyradiculoneuropathy (CIDP) is a rare and heterogeneous immune-mediated neuropathy, usually causing progressive weakness of the extremities with sensory dysfunction. CIDP can lead to severe disability if left untreated [1–3]. The diagnosis of CIDP is based on the European Federation of Neurological Societies/Peripheral Nerve Society in 2010 (EFNS/PNS) 2010 diagnostic criteria for CIDP [1]. However, the diagnosis of CIDP can be challenging [4, 5]. Intravenous immunoglobulins (IVIg), corticosteroids, and plasma exchange (PE) are considered effective first-line treatments for CIDP [6–9]. Most neurologists use either IVIg or corticosteroids as initial treatment [8, 10, 11]. Both treatments have their own distinct advantages and disadvantages. IVIg often leads to rapid improvement in disability and has a low risk of serious adverse events, but the benefit is usually short-lived requiring long-term repeated infusions which carries a significant economic burden [9, 12–14]. Corticosteroids in varying regimens are effective, possibly act slower, seem more likely to induce remissions, and are much cheaper than IVIg [15–17]. The side effect profile of corticosteroids, however, makes them less suitable for long-term use [18]. Combined treatment may offer the advantages of both treatments [19]. Using data from the International CIDP Outcome Study (ICOS), a prospective observational cohort in three Dutch tertiary neuromuscular centers, we aimed to assess response to treatment, treatment status, remission, and residual symptoms or deficits in treatment-naive patients with CIDP 1 year after the start of treatment.

## Methods

### Study design

The ICOS is a prospective observational cohort study in patients with CIDP that started including patients in November 2015 and is currently ongoing in three large tertiary neuromuscular centers: the Erasmus University Medical Center in Rotterdam (Erasmus MC), the Amsterdam University Medical Centers (Amsterdam UMC) and the Utrecht Medical University Center (UMC Utrecht). The ICOS study protocol has been previously reported [20].

### Study population

We identified eligible patients in the ICOS cohort. Inclusion criteria were: adult treatment-naive patients diagnosed with definite, probable or possible CIDP according to the EFNS/PNS 2010 diagnostic criteria for CIDP, with at least 1 year of follow-up, and treated with either 1) IVIg monotherapy, 2) corticosteroids monotherapy or 3) IVIg and corticosteroids (‘combination treatment’) [1]. Treatment-naïve was defined as never treated at the time of inclusion. Most patients in the combination group have been described in a previous publication of the open-label OPTIC pilot [19]. We excluded patients who participated in the randomized, double-blind, placebo-controlled OPTIC trial, as treatment allocation was unknown [21].

### Treatment protocols

Patients were treated according to local treatment protocols. In the Erasmus MC first-choice treatment was IVIg. IVIg treatment consisted of a loading dose (2 g/kg, over 5 days), followed by a second dose (0.4 g/kg/day, over 1–3 days) if required [22]. Maintenance IVIg treatment (0.4 kg/kg every 2–4 weeks) was started if further IVIg treatment was necessary [22]. In Amsterdam UMC, IVIg monotherapy was started in moderately to severely affected patients or pure motor CIDP and consisted of a loading dose (2 g/kg, over 3–5 days) and maintenance treatment (1 g/kg) every 3 weeks, if required [12, 23]. From 2014 onwards, these patients were preferentially treated with a combination protocol (described elsewhere) consisting of IVIg (loading dose 2 g/kg, maintenance 1 g/kg) combined with intravenous methylprednisolone (IVMP, 1000 mg), every 3 weeks during 18 weeks after which treatment was stopped [19]. IVIg (day dose) and IVMP was administered on a single day in random order. In patients with minor deficits, oral dexamethasone 40 mg once daily during four consecutive days, monthly, for 6 months, was started [15]. Patients who relapsed were started on IVIg maintenance treatment. In the UMC Utrecht induction treatment with IVIg was preferred over corticosteroids. IVIg consisted of a loading dose (2 g/kg, over 5 days), followed by a second loading dose if required. Maintenance IVIg treatment (0.4 kg/kg every 2–4 weeks) was started if further IVIg treatment was necessary. Corticosteroid induction treatment consisted of pulsed dexamethasone in the previously described regimen. In all centers, repeated IVIg treatment was considered in case of improvement followed by subsequent deterioration. In the Erasmus MC and UMC Utrecht, maintenance IVIg dose was increased if required whereas in the Amsterdam UMC, dose was titrated from 1 g/kg to the lowest possible dose. IVIg withdrawal was performed periodically in stable patients without a predefined interval. In all centers, patients were switched from IVIg to corticosteroids, or vice versa, in case of lack of or insufficient treatment effect. PE was used by all centers if there was no response to both IVIg and corticosteroids.

### Data collection

Data on relevant demographics, duration of symptoms, CIDP variant, EFNS/PNS 2010 diagnostic category (‘definite’, ‘probable’ or ‘possible’) were collected at entry. The following outcome measures were assessed during follow-up: the inflammatory Rasch built overall disability scale (I-RODS, range 0–100 on centile score) [24], grip strength (measured using a Martin Vigorimeter in kPa) [25, 26], Medical Research Council (MRC) sum score in 12 muscle groups (range 0–60) [27], Inflammatory Neuropathy Cause and Treatment (INCAT) modified sensory sum score (mISS, range 0–33) [28, 29], presence of neuropathic pain (yes/no), Rasch-modified Fatigue Severity Scale (R-FSS, range 0–21) [30] and EQ-5D-5L [31]. Higher scores on the mISS or R-FSS indicate greater severity of sensory symptoms and fatigue respectively. Treatment data included type of treatment, treatment changes, reason for treatment changes, and side effects. Follow-up visits were scheduled at 3, 6, and 12 weeks, and at 6, 12, 18, and 24 months, with an extended (half) yearly follow-up possible. A detailed description of ICOS data collection is provided in the study protocol [20].

### Study outcomes

Patients were grouped based on initial treatment, as listed under ‘study population’. We assessed the following outcome measures: (a) changes in I-RODS, grip strength, and MRC-sum score between baseline, 6 months, and 1 year, (b) the proportion of patients who improved on treatment at 1 year, (c) the proportion of patients who were still treated at 1 year (‘treatment status’) regardless of treatment response, (d) the proportion of patients who improved on treatment and were untreated at 1 year (‘remission’), (e) proportion of patients with residual symptoms or deficits at 1 year, and (f) adherence to protocol, treatment changes between baseline and 1 year. Definition of improvement was not stated in the ICOS protocol. For this study, an improvement on treatment was defined as an increase of at least the minimal clinically important difference (MCID) on the I-RODS [24, 32], ≥ 8 kPa on average grip strength of both hands (measured using a Martin Vigorimeter) [25, 26, 33] and/or ≥ 2 points on the MRC sum score between baseline and 1 year. An MCID related standard error (SE), (MCID-SE), ≥ -1.96 on the I-RODS was considered a relevant clinical improvement [32]. In patients with clinical asymmetric CIDP variant with unilaterally reduced grip strength, increase of ≥ 8 kPa in the affected arm was considered an improvement. We considered grip strength values above the 5th centile for that age group as normal values for quantitative grip strength assessment with the Martin Vigorimeter [25]. Residual symptoms or deficits were defined as any persisting disability or impairment on the I-RODS, grip strength (below age-appropriate normal values), MRC sum score, mISS, R-FSS, EQ-5D-5L domains and the presence of neuropathic pain (pain quality judged by clinician, scored as present or absent) at 1 year. Treatment changes were defined as a change from one treatment type to the other (i.e. IVIg to corticosteroids).

### Statistical analysis

Treatment response, treatment status, and remission rates were compared between the three treatment groups. Scores on the I-RODS, were converted to their equivalent centile metric for analysis [24]. Continuous data are presented as median values with ranges (interquartile range (IQR)). Dichotomous or categorical data are presented as numbers with proportions. We used the Kruskal–Wallis test to compare continuous data, and the Chi-square test to compare proportions. A two-sided *P*-value < 0.05 was considered significant. *P*-values reflect the comparison of the three initial treatment groups, unless stated otherwise. In the case of small numbers per treatment group, no test on significant differences was performed. Correlations between the I-RODS and MRC sum score with the presence of pain (yes/no) and the R-FSS was assessed post hoc using Spearman’s *ρ* correlation coefficient. Correlations between the I-RODS and MRC sum score with the R-FSS were visualized graphically using scatter plots. We used SPSS Statistics version 25.0 for data analysis.

## Results

### Study cohort

By May 2020, 253 patients were included in ICOS. Eleven patients were excluded due to an alternative diagnosis, and a further 202 excluded from the present study for the following reasons: not treatment-naive (*n* = 165), follow-up < 1 year (*n* = 21), participation in the OPTIC trial (*n* = 8), never treated (*n* = 6) or not fulfilling the electrodiagnostic criteria for CIDP (*n* = 2), resulting in 40 patients included for analysis. The median age was 59 (49–68), 32 (76%) patients were male and median symptom duration was 11 (4–42) months (Table 1). Twenty-eight (72%) patients were classified as ‘typical’ CIDP. Eighteen (45%) patients initially received IVIg, 6 (15%) corticosteroids, and 16 (40%) combination treatment. All patients in the corticosteroid monotherapy group received oral pulsed dexamethasone. Age at baseline, gender, and symptom duration did not differ between treatment groups. Patients treated with IVIg were classified as ‘typical’ CIDP more often compared to patients treated with corticosteroids or both, although this was not significantly different. Patients treated with corticosteroids were less affected at baseline than patients treated with IVIg, but differences were not statistically significant.

**Table 1:** Baseline characteristics

	All treatment groups (*n* = 40)	IVIg (*n* = 18)	Corticosteroids^a^ (*n* = 6)	Combination treatment^b^ (*n* = 16)	*p* Value
Demographics					
Age at baseline, median (IQR)	59 (49–68)	60 (46–66)	59 (46–71)	60 (51–69)	0.88
Male, (*n*)	76% (32)	72% (13)	100% (6)	69% (11)	0.30
Symptom duration^c^, months, median (IQR)	11 (4–42)	9 (4–49)	36 (30–61)	10 (3–41)	0.15
Outcome measures					
I-RODS^d^, median (IQR)	60 (42–71)	57 (41–72)	69 (58–73)	56 (43–69)	0.44
Grip strength^e^, median (IQR)	51 (35–78)	48 (31–87)	74 (59–86)	49 (30–63)	0.16
MRC sum score, median (IQR)	54 (50–58)	52 (50–57)	56 (54–60)	54 (48–59)	0.27
mISS, median sum score (IQR)	7 (4–12)	8 (4–16)	5 (3–6)	8 (4–10)	–
Neuropathic pain	35% (14)	39% (7)	50% (3)	25% (4)	0.49
R-FSS, median sum score (range)	16 (10–20)	16 (6–20)	16 (7–21)	16 (10–20)	0.97
EQ-5D-5L					
Any problems mobility, *n*/*N* Any problems self-care, *n*/*N* Any problems usual activities, *n*/*N* Any problems pain/discomfort, *n*/*N* Any problems anxiety/depression, *n*/*N* EQ VAS (IQR)	94% (32/34) 59% (20/34) 88% (30/34) 82% (28/34) 53% (18/34) 63 (48–76)	92% (12/13) 62% (8/13) 92% (12/13) 92% (12/13) 54% (7/13) 60 (40–70)	100% (5/5) 40% (2/5) 100% (5/5) 40% (2/5) 20% (1/5) 65 (55–75)	94% (15/16) 63% (10/16) 81% (13/16) 88% (14/16) 63% (10/16) 63 (43–84)	– – – – – –
EFNS/PNS diagnostic criteria					
EFNS/PNS clinical criteria, typical (*n*)	72% (28)	83% (15)^f^	67% (4)^g^	56% (9)^h^	0.22
EFNS/PNS electrodiagnostic criteria, definite (*n*)	70% (28)	67% (12)	50% (3)	81% (13)	0.33
EFNS/PNS categories criteria, (*n*)					
Definite Probable Possible	83% (33) 10% (4) 8% (3)	83% (15) 11% (2) 6% (1)	83% (5) 0% (0) 17% (1)	81% (13) 13% (2) 6% (1)	NA NA NA

*IVIg* immunoglobulins, *IQR* interquartile range, *I-RODS* inflammatory Rasch built overall disability scale, *MRC* Medical Research Council, *mISS* Inflammatory neuropathy cause and treatment (INCAT) modified sensory sum score, *R-FSS* Rasch-modified Fatigue Severity Scale, *EFNS/PNS* European Federation of Neurological Societies/Peripheral Nerve Society

^a^Pulsed high-dosed dexamethasone

^b^IVIg and methylprednisolone

^c^Symptom duration = time between disease onset and baseline visit

^d^Centiles

^e^Measured with the Martin Vigorimeter

^f^Atypical: asymmetric (*n* = 2), sensory ataxia (*n* = 1)

^g^Atypical: predominantly sensory (*n* = 1), predominantly distal (*n* = 1)

^h^Atypical: predominantly motor (*n* = 3), asymmetric (*n* = 4)

### Treatment response, treatment status, and remission

MCID based improvement on at least one of the outcome measures was seen in 31 (78%) patients; 19 (48%) patients improved on the I-RODS, 28 (70%) on grip strength, and 22 (55%) on the MRC sum score.

Improvement on at least one of the outcome measures was seen in 16 (89%) patients in the IVIg group, 3 (50%) in the corticosteroids group and 12 (75%) in the combination treatment group (*p* = 0.06). Improvement on all three outcome measures was seen in 7 (39%) patients in the IVIg group, 2 (33%) in the corticosteroids group and 5 (31%) in the combination group (*p* = 0.89).

**Fig. 1:**
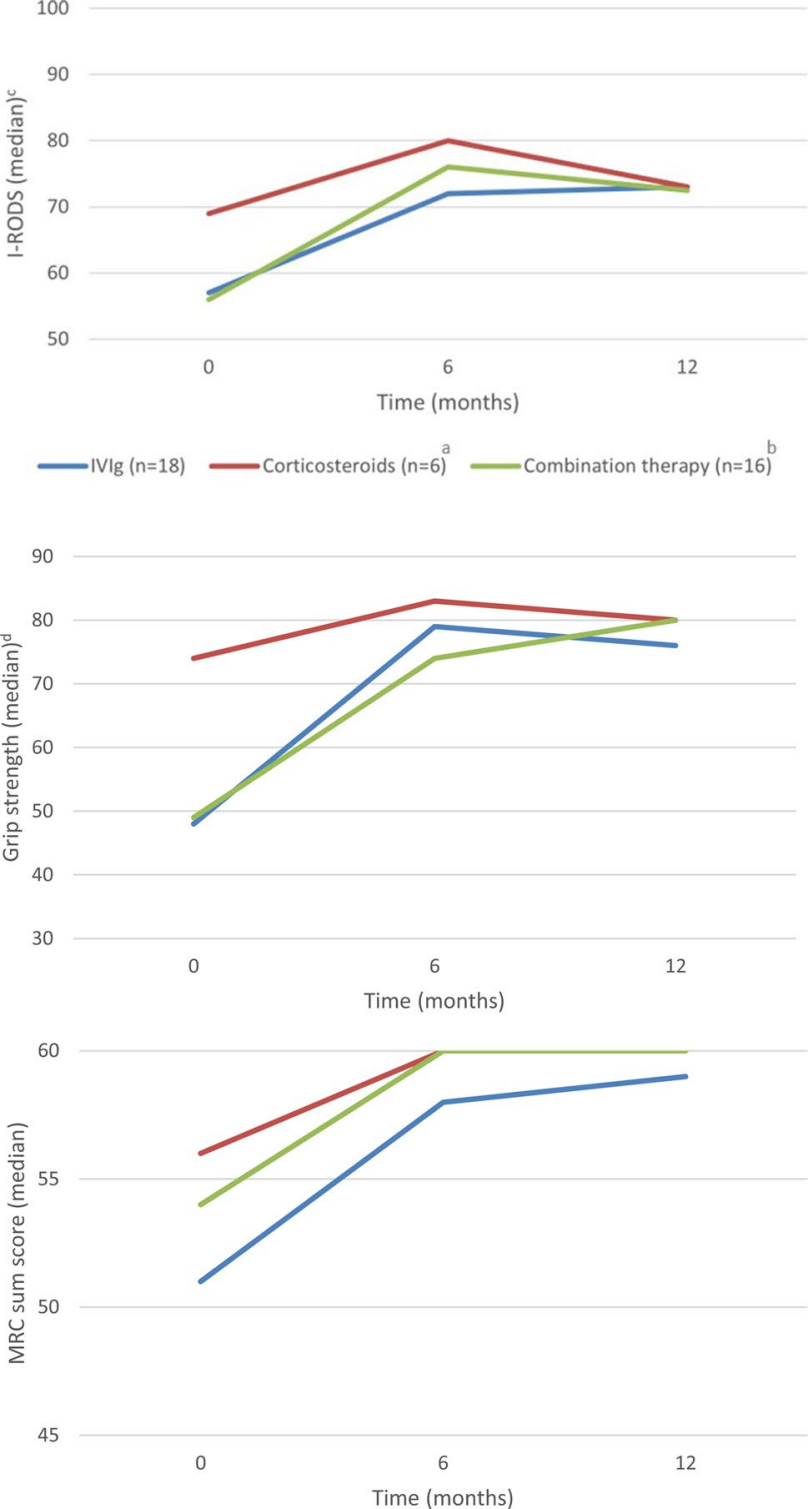
I-RODS, grip strength and MRC-sum score per treatment group during 1 year follow-up. *I-RODS* inflammatory Rasch built overall disability scale, *MRC* Medical Research Council, *IVIg* intravenous immunoglobulins. ^a^Pulsed high-dosed dexamethasone, ^b^IVIg and methylprednisolone, ^c^Centiles, ^d^Measured with the Martin Vigorimeter (kPa)

Changes in I-RODS, grip strength, and MRC-sum score per treatment group are shown in Fig. 1.

**Fig. 2:**
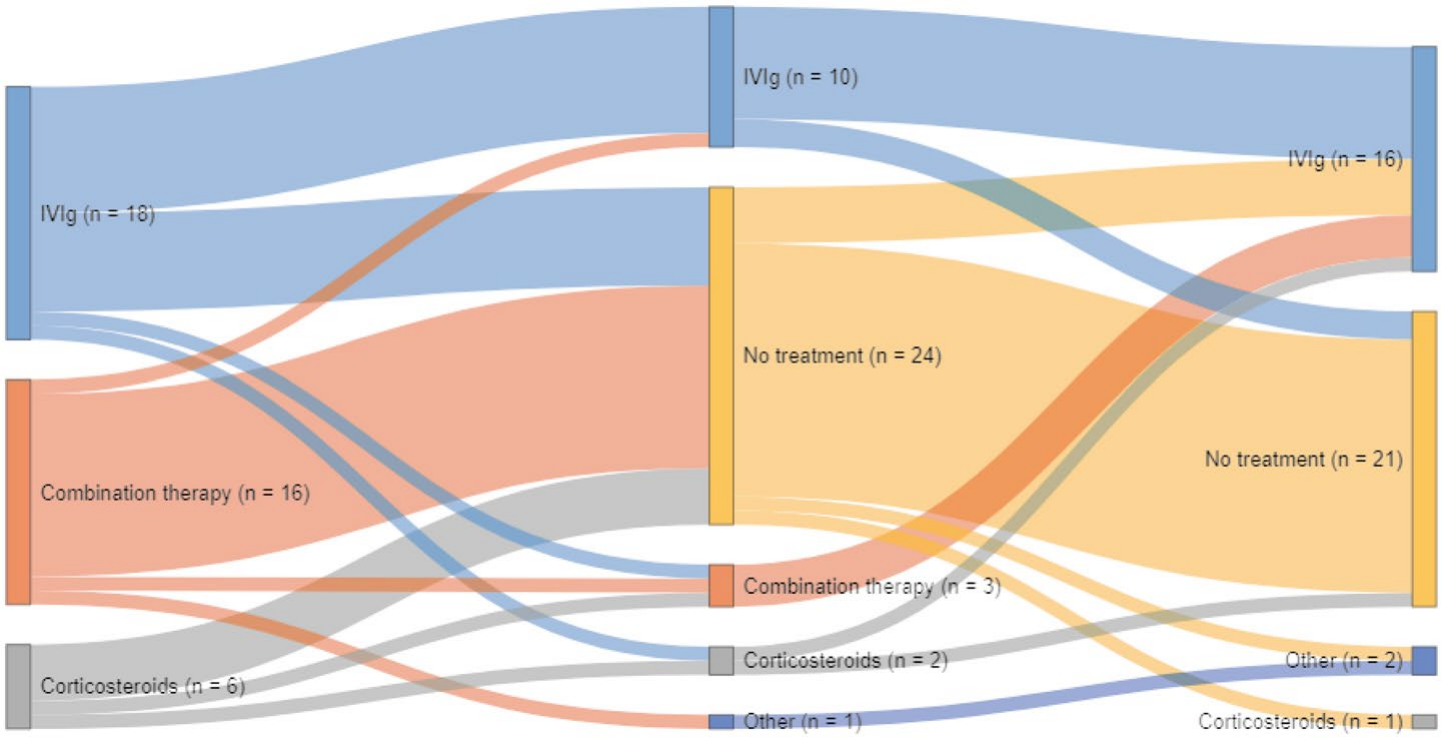
Treatment status and changes during 1 year follow-up. This figure illustrates initial treatment and treatment changes during the course of follow-up. Other at 6 months: PE (*n* = 1). Other at 12 months: SCIg (*n* = 1), PE and methotrexate (*n* = 1). *IVIg* intravenous immunoglobulins; *SCIg* subcutaneous immunoglobulins; *PE* plasma exchange

At one year follow-up, 19 (48%) patients were still treated: 11 (61%) patients in the IVIg group, 2 (33%) in the corticosteroids group, and 6 (38%) in the combination treatment group (p = 0.29, Fig. 2). Of these, 16 were treated with IVIg, one with prednisone, one with SCIg, and one with PE and methotrexate. Two patients were still treated at one year despite not reaching the criteria for improvement. Remission based on improvement on at least one of the outcome measures was seen in 14 patients (36%): 5 (29%) patients in the IVIg group, 2 (33%) in the corticosteroids group and 7 (44%) in the combination treatment group (p = 0.69).

### Residual symptoms and deficits at 1 year

At 1 year follow-up, 34/39 (87%) patients reported at least one activity with difficulty on the I-RODS (median centiles 73; IQR 60–88), 18 (45%) had at least some degree of weakness (median MRC-SS 60; IQR 55–60), 26/28 (93%) had sensory deficits (median mISS 7, IQR 2–8), 10 (25%) had neuropathic pain, and 26/29 (90%) reported fatigue (median R-FSS sum score 13; IQR 5–15) (Table 2). Eighteen out of 30 (60%) patients reported problems with mobility, 8/30 (27%) with self-care, 19/30 (63%) with usual activities, 21/30 (70%) with pain and/or discomfort, and 8/32 (27%) with anxiety and/or depression (Table S1). Lower I-RODS scores were associated with higher R-FSS scores (Spearman’s *ρ* = − 0.74; *p* < 0.01; Figure S1) and not associated with pain (Spearman’s *ρ* = − 0.17; *p* = 0.31). Lower MRC sum scores were not associated with higher R-FSS scores (Spearman’s *ρ* = − 0.23; *p* = 0.23; Figure S2*)* and pain (Spearman’s *ρ* = − 0.12; *p* = 0.46).

**Table 2:** Residual symptoms and deficits at 1 year (*n* = 40)

	All treatment groups (*n* = 40)	IVIg (*n* = 18)	Corticosteroids^a^ (*n* = 6)	Combination treatment^b^ (*n* = 16)	Treated at 1 year (*n* = 19)	Untreated at 1 year (*n* = 21)
I-RODS						
Median centiles (IQR) Any problems on I-RODS, % (*n*/*N*)	73 (60–88) 87 (34/39)	73 (67–86) 88 (15/17)	73 (52–89) 100 (6/6)	73 (56–92) 81 (13/16)	76 (56–89) 83 (15/18)	71 (62–88) 90 (19)
Grip strength						
Median kPa (IQR) Weakness grip strength, % (*n*)	78 (61–94) 45 (18)	76 (60–93) 56 (10)	80 (69–107) 33 (2)	80 (60–93) 38 (6)	77 (65–93) 47 (9)	79 (59–94) 43 (9)
MRC-SS						
Median sum score (IQR) Any weakness^a^ on MRC-SS, % (*n*)	60 (55–60) 45 (18)	59 (54–60) 56 (10)	60 (59–60) 17 (1)	60 (56–60) 44 (7)	60 (54–60) 47 (9)	60 (56–60) 43 (9)
mISS						
Median sum score (IQR) Any sensory deficits on mISS, % (*n*/*N*)	7 (2–8) 93 (26/28)	8 (5–11) 93 (14/15)	8 (-) 100 (3/3)	4 (1–6) 90 (9/10)	8 (1–10) 83 (10/12)	6 (3–8) 100 (16/16)
Neuropathic pain						
Presence of neuropathic pain, % (*n*)	25 (10)	33 (6)	33 (2)	13 (2)	37 (7)	14 (3)
R-FSS						
Median sum score (IQR) Any fatigue on R-FSS, % (*n*/*N*)	13 (5–15) 90 (26/29)	14 (6–15) 91 (10/11)	10 (4–15) 100 (4/4)	13 (5–18) 86 (12/14)	13 (6–16) 93 (13/14)	13 (4–15) 87 (13/15)
EQ-5D-5L						
Any problems mobility, % (*n*/*N*) Any problems self-care, % (*n*/*N*) Any problems usual activities, % (*n*/*N*) Any problems pain/discomfort, % (*n*/*N*) Any problems anxiety/depression, % (*n*/*N*) Any problems total, % (*n*/*N*) EQ VAS (IQR)	60 (18/30) 27 (8/30) 63 (19/30) 70 (21/30) 27 (8/30) 93 (28/30) 78 (65–85)	62 (8/13) 15 (2/13) 69 (9/13) 69 (9/13) 15 (2/13) 92 (12/13) 78 (68–80)	50 (2/4) 25 (1/4) 25 (1/4) 75 (3/4) 50 (2/4) 100 (4/4) 80 (73–91)	62 (8/13) 39 (5/13) 69 (9/13) 69 (9/13) 31 (4/13) 92 (12/13) 73 (60–90)	63 (10/16) 13 (2/16) 56 (9/16) 69 (11/16) 19 (3/16) 88 (14/16) 75 (61–84)	57 (8/14) 43 (6/14) 71 (10/14) 71 (10/14) 36 (5/14) 100 (14/14) 80 (65–85)

*IVIg* immunoglobulins, *IQR* interquartile range, *I-RODS* inflammatory Rasch built overall disability scale, *kPa* kilopascal, *MRC-SS* Medical Research Council Sum Score, *mISS* inflammatory neuropathy cause and treatment (INCAT) modified sensory sum score, *R-FSS* Rasch-modified Fatigue Severity Scale, *EQ VAS* visual analogue scale

^a^Based on 0.05 quantile values per age span [23]

^a^Pulsed high-dosed dexamethasone

^b^IVIg and methylprednisolone

### Adherence to treatment protocol and treatment changes

In the IVIg group, treatment was changed in two patients, due to inefficacy or side effects (Fig. 2). In the corticosteroids group, two patients (33%) completed the treatment protocol. The remaining four (67%) patients received between 3 and 5 dexamethasone courses. Side effects (mood changes, insomnia and fatigue) factored into the decision not to complete all six courses in all four patients. In the combination group, two patients (13%) did not complete the protocol [19].

### Treatment withdrawal and relapses

In the IVIg group, five patients (28%) received between one and three loading doses IVIg (2 g/kg) only. Three of these five patients improved and did not require maintenance treatment. The remaining two patients did not improve but only had mild complaints and did not receive further treatment. Thirteen patients (72%) received IVIg maintenance therapy (25–50 g, every 2–4 weeks). In 6/13 (46%) of patients on IVIg maintenance treatment, a withdrawal attempt was performed in the first year, of which four relapsed. Time to relapse ranged from 1 to 13 weeks. There was no difference between the IVIg dose and duration of IVIg treatment prior to IVIg withdrawal attempt between those who relapsed and those who did not (data not shown). One patient (17%) in the corticosteroid group relapsed two months after finishing his last course. Five of 14 (36%) patients in the combination group that completed the protocol, experienced a relapse. Time to relapse was 3 months after stopping treatment in the corticosteroid monotherapy group and ranging from 3 weeks to 7 months after finishing treatment in the combination group.

## Discussion

Improvement on at least one of the clinical outcome measures occurred in a majority of patients, with improvement captured most frequently on grip strength. Overall, improvement was observed most frequently in the IVIg group, but this was not statistically significant. In the whole group, slightly more than half of treatment-naive patients were untreated at 1 year and around one-third were in remission. Despite treatment and an objective treatment response in most patients, the majority of patients reported residual symptoms or deficits at one year.

Defining improvement in CIDP remains a matter of debate as currently used MCID’s do not always represent clinically important change. Changes that are smaller than the MCID might be clinically relevant in some cases, especially if consistent over time and if measured on different outcome measures. However, it should be emphasized that in most responders to treatment, improvement was robust and seen on more than one outcome measure.

In this study, most patients started on IVIg improved across all outcome measures, with improvement captured most frequently on grip strength. Improvement rates in patients started on IVIg were similar to those reported in the literature [12, 14, 34]. In the corticosteroid monotherapy group, improvement was found in 33–50% of patients depending on the outcome measure. This is somewhat lower or comparable to previous reports in the literature, in which improvement rates range from 48 to 54% [15, 16, 35, 36]. Patients treated with corticosteroid monotherapy tended to have a lower degree of baseline disability on all clinical outcome measures, reflecting the selection of patients as pre-specified in the Amsterdam UMC protocol. This relatively lower degree of baseline disability potentially leaves less room for improvement (‘ceiling effect’). In addition, patients in the corticosteroid monotherapy group reported a longer duration of symptoms at inclusion. This may point to a more indolent disease course where a delayed initiation of treatment may have led to (irreversible) axonal damage. Some patients who did not meet our criteria for improvement did not receive additional treatment, remaining stable during the course of follow-up. In patients with relatively minor disability and no initial treatment response, a wait and see approach may be considered to determine the presence of active disease.

Less than one-third of patients started on IVIg were in remission at 1 year, while most of the other patients that started on IVIg were still receiving maintenance treatment. A similar proportion of patients started on IVIg only received one or more loading doses and did not receive maintenance treatment, comparable to the 15–30% range described in the literature [1, 34]. IVIg withdrawal was performed in less than half of patients who received IVIg maintenance therapy, contributing to the low frequency of remissions in the IVIg group according to our definition. We were unable to determine whether different maintenance doses in the IVIg group contributed to the likelihood of being untreated at 1 year. The majority of patients in whom withdrawal was attempted relapsed, which is within the broad range (38–89%) of reported IVIg dependency in the literature [16, 34, 36, 37]. The infrequency of withdrawal attempts in our cohort may reflect the relatively short follow-up. Some patients may not have improved sufficiently, may not have been considered clinically stable or exhibited end of dose symptoms. Ideally, withdrawal should be attempted in patients even in their first year of maintenance treatment to assess IVIg dependency [38]. One-third of patients treated with corticosteroid monotherapy were in remission and most were untreated at 1 year. Few studies using (varying) regimens of pulsed corticosteroids suggest that patients treated with corticosteroids can reach clinical remission for a prolonged period of time [17, 19, 39]. In the combined treatment group, most patients were untreated at 1 year and slightly less than half was in remission at that time. A randomized controlled trial (OPTIC trial) is currently underway to determine whether combined treatment safely leads to more frequent remissions compared to IVIg treatment alone [21]. Treatment was changed in only one patient in the IVIg group due to side effects, illustrating the tolerability for IVIg [9, 14, 34]. Side effects led to a temporary stop of treatment and factored into the decision to stop treatment earlier in a considerable number of patients in the corticosteroid monotherapy group. Counseling prior to the start of treatment, along with careful monitoring of improvement and side effects, should constitute an essential part of this treatment regimen.

Residual symptoms and deficits were found to a variable extent on all outcome measures. Only a few patients did not report any disability on the I-RODS and in almost half of the patients examined at 1 year, grip strength and muscle weakness persisted. Residual sensory symptoms or deficits were found in nearly all patients at 1 year. A considerable number of patients reported neuropathic pain and nearly all patients reported fatigue interfering with daily activities. Both fatigue and pain are common (long-term) symptoms and can persist despite treatment [40–43]. The reported median fatigue scores in our study are comparable to other data in the literature and considerably higher than median scores derived from control populations as reported in other studies [41, 42]. The proportion of patients reporting neuropathic pain is also comparable to values reported in other prospective studies [43] In the patients reporting neuropathic pain, the CIDP diagnosis remained unchanged. Less patients reported neuropathic pain at 1 year than before the start of treatment and fatigue scores were lower. Many patients in whom no muscle weakness was found at 1 year, reported residual symptoms and deficits. A lower I-RODS score, indicating more disability, was associated with more fatigue, but no other association with fatigue and pain was identified. Overall health at 1 year was rated higher than at baseline, and only slightly lower than that of the general Dutch population (EQ VAS score of 78 versus 80) in the appropriate age reference group (55–64 years) [44]. This shows that despite residual symptoms and deficits, the overall quality of life is highly rated. All EQ-5D-5L modalities showed a decrease in patients reporting problems at 1 year. A considerable number of patients reported problems on the individual domains, in line with previous literature [45, 46]. Although pain and fatigue can be also attributed to active disease, we would like to emphasize that residual symptoms on themselves should not be the reason to start or continue immunomodulating or immunosuppressive treatment in absence of objective signs of active disease. Our definition of residual symptoms may overestimate their prevalence and confounders, such as age and other comorbidities, may influence these results. Treating physicians should consider referring patients with residual deficits to rehabilitation specialists, physicals and occupational therapists, or pain specialists for supportive care.

Strengths of this study on real-world data are the focus on treatment-naïve patients, the prospective multicenter design, and the relatively long follow-up. Prospective data were not limited to accepted clinical disability and impairment outcomes, but also included important outcome measures such as pain, fatigue, additional support and quality of life assessments that are infrequently described in a prospective nature in the literature [47, 48]. The sample size was too small to allow comparison between treatment groups. Moreover, variability in treatment protocols between the three centers and changes in treatment contributed to the different patient characteristics and sample sizes across treatment groups that may have introduced bias. However, this also reflects the normal variation in routine clinical practice. The setting of three tertiary neuromuscular centers may have also introduced bias and may impact the generalizability of the results. For these reasons, comparison between treatment groups was not the main objective of this study and any differences found in this study should be interpreted with caution. Clear and uniform management guidelines on when and how to evaluate treatment response, attempt treatment withdrawal, and re-initiate treatment are warranted to further optimize treatment across different centers worldwide. Our study used three different outcome measures along with MCID based cut-off values to define improvement. We recognize that these criteria do not always reflect clinical practice and that a less static, more patient-tailored assessment may be more suitable to define meaningful improvement and treatment (non) responders [47, 49–51]. We advocate a multimodal approach in those patients with expected limited improvement, for example in patients with severe axonal damage at presentation or those with only minimal disability or impairment, if treatment is considered justified at all. When considering improvement, consistency in measurements in these patients with small changes is vital [51]. The lack of a control group with age-matched healthy controls is another limitation, as it makes it inherently difficult to assess whether all (residual) symptoms can be contributed to CIDP. Finally, we assessed remission at 1 year regardless of the duration of the treatment-free period. Longer term studies are needed to determine whether the outcomes evaluated in this study are maintained for a longer period of time.

## Conclusions

Improvement was seen in most treatment-naïve patients and occurred most frequently in CIDP patients started on IVIg treatment. Slightly more than half of treatment-naive CIDP patients were untreated with around one-third in remission at 1 year. Patients in the combination treatment group showed the highest percentage of remissions. Comparability of clinical outcomes between treatment groups is limited due to the small numbers of patients per treatment group and potential selection bias. Residual symptoms and deficits were present in a considerable number of patients, regardless of which treatment was started.

## Supplementary Information

Below is the link to the electronic supplementary material.

Supplementary file1 (DOCX 59 KB)

## Data Availability

Data may be shared upon reasonable request by any qualified investigator.

## References

[1] Van den Bergh PY, Hadden RD, Bouche P, Cornblath DR, Hahn A, Illa I, Koski CL, Leger JM, Nobile-Orazio E, Pollard J, Sommer C, van Doorn PA, van Schaik IN, European Federation of Neurological S, Peripheral Nerve S (2010). European Federation of Neurological Societies/Peripheral Nerve Society guideline on management of chronic inflammatory demyelinating polyradiculoneuropathy: report of a joint task force of the European Federation of Neurological Societies and the Peripheral Nerve Society—first revision. Eur J Neurol.

[2] Broers MC, Bunschoten C, Nieboer D, Lingsma HF, Jacobs BC (2019). Incidence and prevalence of chronic inflammatory demyelinating polyradiculoneuropathy: a systematic review and meta-analysis. Neuroepidemiology.

[3] Lehmann HC, Burke D, Kuwabara S (2019). Chronic inflammatory demyelinating polyneuropathy: update on diagnosis, immunopathogenesis and treatment. J Neurol Neurosurg Psychiatry.

[4] Eftimov F, Lucke IM, Querol LA, Rajabally YA, Verhamme C (2020). Diagnostic challenges in chronic inflammatory demyelinating polyradiculoneuropathy. Brain.

[5] Stino AM, Naddaf E, Dyck PJ, Dyck PJB (2021). Chronic inflammatory demyelinating polyradiculoneuropathy-Diagnostic pitfalls and treatment approach. Muscle Nerve.

[6] Hughes RA, Mehndiratta MM (2015). Corticosteroids for chronic inflammatory demyelinating polyradiculoneuropathy. Cochrane Database Syst Rev.

[7] Mehndiratta MM, Hughes RA, Pritchard J (2015). Plasma exchange for chronic inflammatory demyelinating polyradiculoneuropathy. Cochrane Database Syst Rev.

[8] Oaklander AL, Lunn MP, Hughes RA, van Schaik IN, Frost C, Chalk CH (2017). Treatments for chronic inflammatory demyelinating polyradiculoneuropathy (CIDP): an overview of systematic reviews. Cochrane Database Syst Rev.

[9] Eftimov F, Winer JB, Vermeulen M, de Haan R, van Schaik IN (2013). Intravenous immunoglobulin for chronic inflammatory demyelinating polyradiculoneuropathy. Cochrane Database Syst Rev.

[10] Gelinas D, Katz J, Nisbet P, England JD (2019). Current practice patterns in CIDP: a cross-sectional survey of neurologists in the United States. J Neurol Sci.

[11] Broers MC, van Doorn PA, Kuitwaard K, Eftimov F, Wirtz PW, Goedee S, Lingsma HF, Jacobs BC (2020). Diagnosis and treatment of chronic inflammatory demyelinating polyradiculoneuropathy in clinical practice: a survey among Dutch neurologists. J Peripher Nerv Syst.

[12] Hughes RAC, Donofrio P, Bril V, Dalakas MC, Deng C, Hanna K, Hartung H-P, Latov N, Merkies ISJ, van Doorn PA (2008). Intravenous immune globulin (10% caprylate-chromatography purified) for the treatment of chronic inflammatory demyelinating polyradiculoneuropathy (ICE study): a randomised placebo-controlled trial. Lancet Neurol.

[13] Latov N, Deng C, Dalakas MC, Bril V, Donofrio P, Hanna K, Hartung HP, Hughes RA, Merkies IS, van Doorn PA, Group I-CCES (2010). Timing and course of clinical response to intravenous immunoglobulin in chronic inflammatory demyelinating polyradiculoneuropathy. Arch Neurol.

[14] Kuwabara S, Mori M, Misawa S, Suzuki M, Nishiyama K, Mutoh T, Doi S, Kokubun N, Kamijo M, Yoshikawa H, Abe K, Nishida Y, Okada K, Sekiguchi K, Sakamoto K, Kusunoki S, Sobue G, Kaji R, Glovenin ICSG (2017). Intravenous immunoglobulin for maintenance treatment of chronic inflammatory demyelinating polyneuropathy: a multicentre, open-label, 52-week phase III trial. J Neurol Neurosurg Psychiatry.

[15] van Schaik IN, Eftimov F, van Doorn PA, Brusse E, van den Berg LH, van der Pol WL, Faber CG, van Oostrom JCH, Vogels OJM, Hadden RDM, Kleine BU, van Norden AGW, Verschuuren JJGM, Dijkgraaf MGW, Vermeulen M (2010). Pulsed high-dose dexamethasone versus standard prednisolone treatment for chronic inflammatory demyelinating polyradiculoneuropathy (PREDICT study): a double-blind, randomised, controlled trial. Lancet Neurol.

[16] Nobile-Orazio E, Cocito D, Jann S, Uncini A, Beghi E, Messina P, Antonini G, Fazio R, Gallia F, Schenone A, Francia A, Pareyson D, Santoro L, Tamburin S, Macchia R, Cavaletti G, Giannini F, Sabatelli M (2012). Intravenous immunoglobulin versus intravenous methylprednisolone for chronic inflammatory demyelinating polyradiculoneuropathy: a randomised controlled trial. Lancet Neurol.

[17] Nobile-Orazio E, Cocito D, Jann S, Uncini A, Messina P, Antonini G, Fazio R, Gallia F, Schenone A, Francia A, Pareyson D, Santoro L, Tamburin S, Cavaletti G, Giannini F, Sabatelli M, Beghi E, Group IMCT (2015). Frequency and time to relapse after discontinuing 6-month therapy with IVIg or pulsed methylprednisolone in CIDP. J Neurol Neurosurg Psychiatry.

[18] Oray M, Abu Samra K, Ebrahimiadib N, Meese H, Foster CS (2016). Long-term side effects of glucocorticoids. Expert Opin Drug Saf.

[19] Adrichem ME, Bus S, Wieske L, Mohammed H, Verhamme C, Hadden R, van Schaik IN, Eftimov F (2019). Combined intravenous immunoglobulin and methylprednisolone as induction treatment in chronic inflammatory demyelinating polyneuropathy (OPTIC-protocol); a prospective pilot study. Eur J Neurol.

[20] Bunschoten C, Eftimov F, van der Pol WL, Jacobs BC, Consortium I (2019). International chronic inflammatory demyelinating polyneuropathy outcome study (ICOS): Protocol of a prospective observational cohort study on clinical and biological predictors of disease course and outcome. J Peripher Nerv Syst.

[21] ISRCTN15893334 (2018). Intravenous immunoglobulin and intravenous methylprednisolone as optimal first line treatment in chronic inflammatory demyelinating polyneuropathy (CIDP).

[22] Kuitwaard K, Fokkink WJR, Brusse E, Vrancken AFJE, Eftimov F, Notermans NC, van der Kooi AJ, Merkies ISJ, Jacobs BC, van Doorn PA (2017). Maintenance IV immunoglobulin treatment in chronic inflammatory demyelinating polyradiculoneuropathy. J Peripher Nerv Syst.

[23] Eftimov F, van Schaik IN (2012). De behandeling van chronische inflammatoire demyeliniserende polyradiculoneuropathie. Tijdschrift voor Neurologie en Neurochirurgie.

[24] van Nes SI, Vanhoutte EK, van Doorn PA, Hermans M, Bakkers M, Kuitwaard K, Faber CG, Merkies IS (2011). Rasch-built Overall Disability Scale (R-ODS) for immune-mediated peripheral neuropathies. Neurology.

[25] Merkies IS, Schmitz PI, Samijn JP, Meche FG, Toyka KV, van Doorn PA (2000). Assessing grip strength in healthy individuals and patients with immune-mediated polyneuropathies. Muscle Nerve.

[26] Vanhoutte EK, Latov N, Deng C, Hanna K, Hughes RA, Bril V, Dalakas MC, Donofrio P, van Doorn PA, Hartung HP, Merkies IS (2013). Vigorimeter grip strength in CIDP: a responsive tool that rapidly measures the effect of IVIG–the ICE study. Eur J Neurol.

[27] Kleyweg RP, van der Meche FG, Schmitz PI (1991). Interobserver agreement in the assessment of muscle strength and functional abilities in Guillain-Barre syndrome. Muscle Nerve.

[28] Vanhoutte EK, Faber CG, Merkies IS, PeriNomS study group (2013). 196th ENMC international workshop: outcome measures in inflammatory peripheral neuropathies 8–10 February 2013, Naarden, The Netherlands. Neuromuscul Disord.

[29] Merkies IS, Lauria G (2006). 131st ENMC international workshop: selection of outcome measures for peripheral neuropathy clinical trials 10–12 December 2004, Naarden, The Netherlands. Neuromuscul Disord.

[30] van Nes SI, Vanhoutte EK, Faber CG, Garssen M, van Doorn PA, Merkies IS, PeriNom SSG (2009). Improving fatigue assessment in immune-mediated neuropathies: the modified Rasch-built fatigue severity scale. J Peripher Nerv Syst.

[31] Herdman M, Gudex C, Lloyd A, Janssen M, Kind P, Parkin D, Bonsel G, Badia X (2011). Development and preliminary testing of the new five-level version of EQ-5D (EQ-5D-5L). Qual Life Res.

[32] Draak TH, Vanhoutte EK, van Nes SI, Gorson KC, Van der Pol WL, Notermans NC, Nobile-Orazio E, Leger JM, Van den Bergh PY, Lauria G, Bril V, Katzberg H, Lunn MP, Pouget J, van der Kooi AJ, Hahn AF, Doorn PA, Cornblath DR, van den Berg LH, Faber CG, Merkies IS, PeriNom SSG (2014). Changing outcome in inflammatory neuropathies: Rasch-comparative responsiveness. Neurology.

[33] Merkies IS, van Nes SI, Hanna K, Hughes RA, Deng C (2010). Confirming the efficacy of intravenous immunoglobulin in CIDP through minimum clinically important differences: shifting from statistical significance to clinical relevance. J Neurol Neurosurg Psychiatry.

[34] Kuitwaard K, Hahn AF, Vermeulen M, Venance SL, van Doorn PA (2015). Intravenous immunoglobulin response in treatment-naive chronic inflammatory demyelinating polyradiculoneuropathy. J Neurol Neurosurg Psychiatry.

[35] Hughes R, Bensa S, Willison H, Van den Bergh P, Comi G, Illa I, Nobile-Orazio E, van Doorn P, Dalakas M, Bojar M, Swan A, Inflammatory Neuropathy C, Treatment G (2001). Randomized controlled trial of intravenous immunoglobulin versus oral prednisolone in chronic inflammatory demyelinating polyradiculoneuropathy. Ann Neurol.

[36] Hughes RA, Mehndiratta MM, Rajabally YA (2017). Corticosteroids for chronic inflammatory demyelinating polyradiculoneuropathy. Cochrane Database Syst Rev.

[37] van Schaik IN, Bril V, van Geloven N, Hartung HP, Lewis RA, Sobue G, Lawo JP, Praus M, Mielke O, Durn BL, Cornblath DR, Merkies ISJ, group Ps, (2018). Subcutaneous immunoglobulin for maintenance treatment in chronic inflammatory demyelinating polyneuropathy (PATH): a randomised, double-blind, placebo-controlled, phase 3 trial. Lancet Neurol.

[38] Lunn MP, Ellis L, Hadden RD, Rajabally YA, Winer JB, Reilly MM (2016). A proposed dosing algorithm for the individualized dosing of human immunoglobulin in chronic inflammatory neuropathies. J Peripher Nerv Syst.

[39] Eftimov F, Vermeulen M, van Doorn PA, Brusse E, van Schaik IN, Predict (2012). Long-term remission of CIDP after pulsed dexamethasone or short-term prednisolone treatment. Neurology.

[40] Kuitwaard K, Bos-Eyssen ME, Blomkwist-Markens PH, van Doorn PA (2009). Recurrences, vaccinations and long-term symptoms in GBS and CIDP. J Peripher Nerv Syst.

[41] Merkies IS, Schmitz PI, Samijn JP, van der Meche FG, van Doorn PA (1999). Fatigue in immune-mediated polyneuropathies. European Inflammatory Neuropathy Cause and Treatment (INCAT) Group. Neurology.

[42] Gable KL, Attarian H, Allen JA (2020). Fatigue in chronic inflammatory demyelinating polyneuropathy. Muscle Nerve.

[43] Bjelica B, Peric S, Bozovic I, Kacar A, Cobeljic M, Dejanovic I, Stevic Z, Basta I (2019). One-year follow-up study of neuropathic pain in chronic inflammatory demyelinating polyradiculoneuropathy. J Peripher Nerv Syst.

[44] Janssen B, Szende A, Szende A, Janssen B, Cabases J (2014). Population norms for the EQ-5D. Self-reported population health: an international perspective based on EQ-5D.

[45] Mahdi-Rogers M, McCrone P, Hughes RA (2014). Economic costs and quality of life in chronic inflammatory neuropathies in southeast England. Eur J Neurol.

[46] Ryltoft AK, Al-Zuhairy A, Sindrup SH, Andersen H, Markvardsen LK (2020). Quality of life in chronic inflammatory demyelinating polyneuropathy patients treated with subcutaneous immunoglobulin. Acta Neurol Scand.

[47] Allen JA, Merkies ISJ, Lewis RA (2020). Monitoring clinical course and treatment response in chronic inflammatory demyelinating polyneuropathy during routine care: a review of clinical and laboratory assessment measures. JAMA Neurol.

[48] Bozovic I, Peric M, Arsic Azanjac A, Palibrk A, Bulatovic I, Aleksic D, Peric S, Basta I (2021). Prospective analysis of disability and quality of life in patients with chronic inflammatory demyelinating polyradiculoneuropathy. Qual Life Res.

[49] Sadjadi R, Peric S, Gwathmey K, Bozovic I, Aleksa P, Bjelica B, Burns T, Basta I (2021). Psychometric longitudinal evaluation of the Chronic Acquired Polyneuropathy Patient-Reported Index (CAPPRI) in patients with chronic inflammatory demyelinating polyneuropathy. Muscle Nerve.

[50] Doneddu PE, Mandia D, Gentile F, Gallia F, Liberatore G, Terenghi F, Ruiz M, Nobile-Orazio E (2020). Home monitoring of maintenance intravenous immunoglobulin therapy in patients with chronic inflammatory neuropathy. J Peripher Nerv Syst.

[51] Doneddu PE, Hadden RDM (2020). Daily grip strength response to intravenous immunoglobulin in chronic immune neuropathies. Muscle Nerve.

