# Supplementary material for: Clinical outcome of CIDP one year after start of treatment: a prospective cohort study
Source: J Neurol. 2021 Jun 26;269(2):945–55. doi: 10.1007/s00415-021-10677-5 (PMC8782785; doi:10.1007/s00415-021-10677-5)
Supplement: Supplementary file 1 — Supplementary file1 (DOCX 59 KB) [file 415_2021_10677_MOESM1_ESM.docx]

**Supplemental tables and figures**

**Table S1. EQ-5D-5L percentages and frequencies reported by dimension and level at 1 year (N=30)**

|  | MOBILITY  (n) | SELF-CARE  (n) | USUAL ACTIVITIES  (n) | PAIN/  DISCOMFORT  (n) | ANXIETY/  DEPRESSION  (n) |
| --- | --- | --- | --- | --- | --- |
| Level 1  (No problems) | 40% (12) | 74% (22) | 37% (11) | 30% (9) | 73% (22) |
| Level 2  (Slight problems) | 23% (7) | 13% (4) | 33% (10) | 40% (12) | 20% (6) |
| Level 3  (Moderate problems) | 27% (8) | 10% (3) | 20% (6) | 27% (8) | 3% (1) |
| Level 4  (Severe problems) | 10% (3) | 3% (1) | 7% (2) | 3% (1) | 3% (1) |
| Level 5  (Extreme problems/  unable to do) | 0% (0) | 0% (0) | 3% (1) | 0% (0) | 0% (0) |

**Figure S1. Scatter plot of the correlation between the I-RODS and R-FSS at 1 year**

**
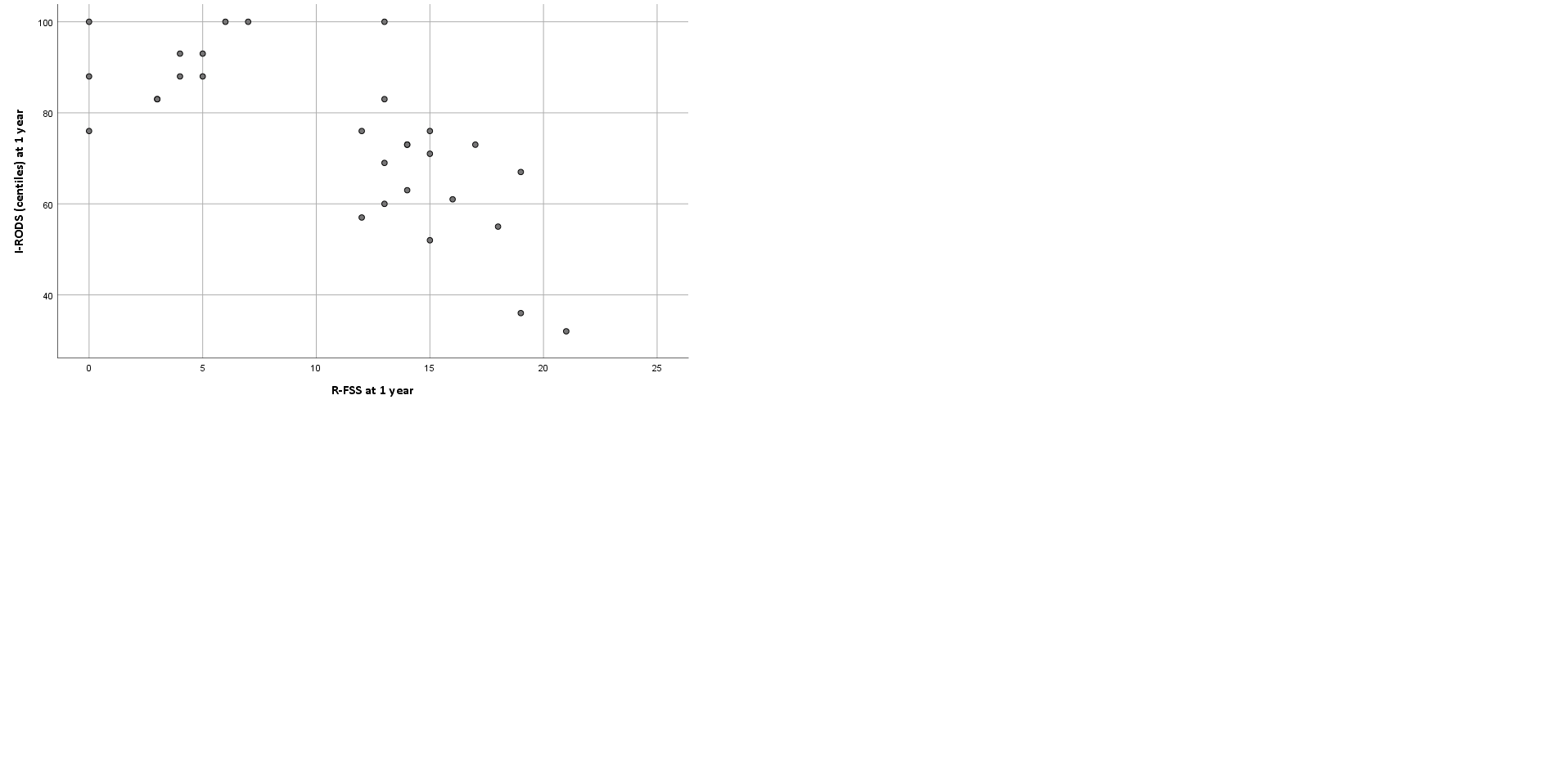
**

Abbreviations: : I-RODS = inflammatory Rasch built overall disability scale, R-FSS = Rasch-modified Fatigue Severity Scale

**Figure S2. Scatter plot of the correlation between the MRC sum score and R-FSS at 1 year**

**
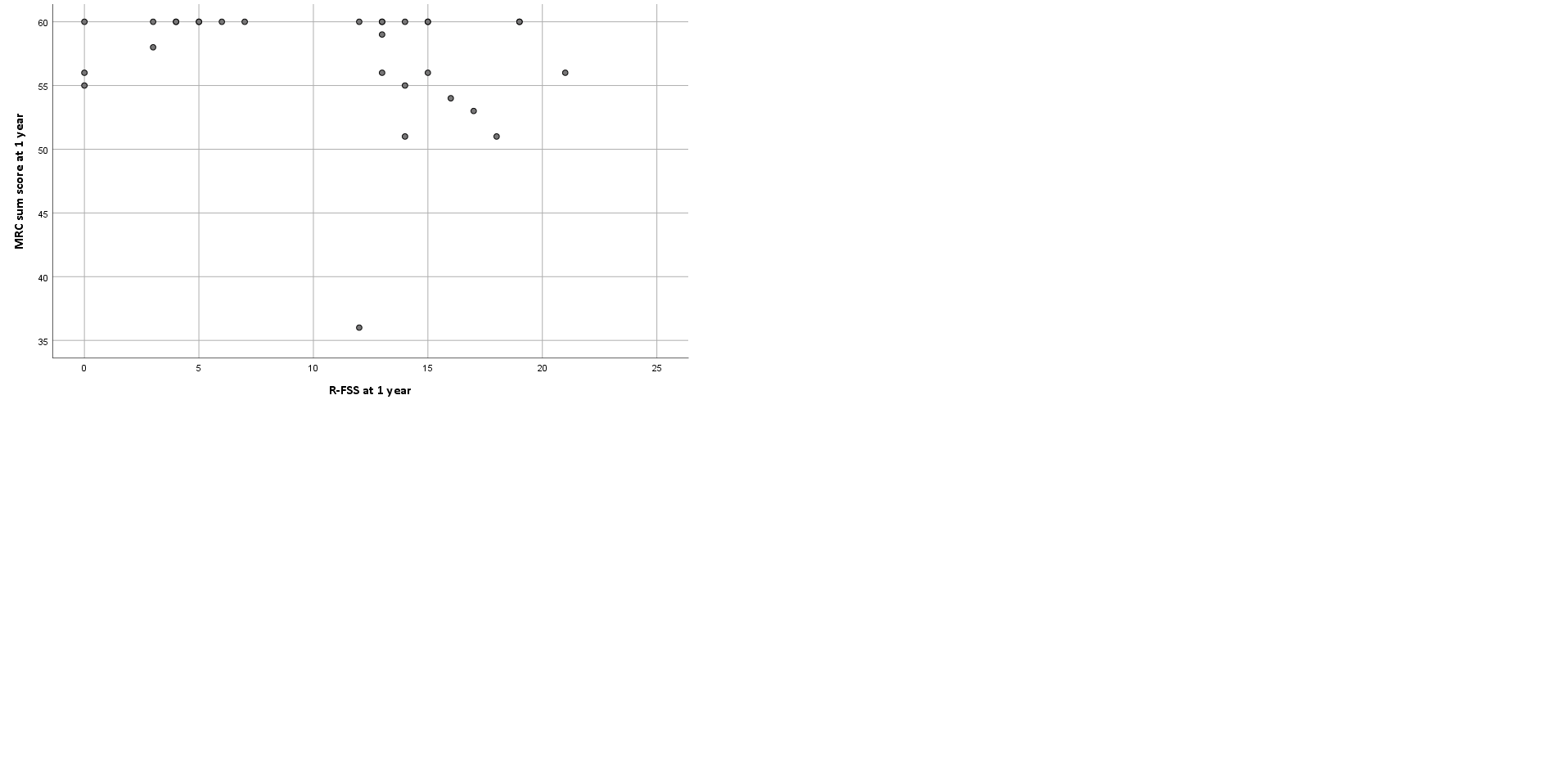
**

Abbreviations: MRC = Medical Research Council, R-FSS = Rasch-modified Fatigue Severity Scale
